# 2-(3-Oxocyclo­hex-1-enylamino)acetic acid

**DOI:** 10.1107/S1600536809039646

**Published:** 2009-10-17

**Authors:** Li Liu, Li-Zhen Liu, Xi-Zheng Liu, Bo Bi, Lin Xu

**Affiliations:** a Key Laboratory of Polyoxometalate Science of the Ministry of Education, Northeast Normal University, Changchun 130024, People’s Republic of China

## Abstract

The six-membered ring of the title compound, C_8_H_11_NO_3_, adopts an envelope shape with the C atom in the *meta* position of the carbonyl representing the flap. This atom is disordered over two positions in an 0.865 (6): 0.135 (6) ratio. In the crystal, a two-dimensional supra­molecular network parallel to the *ac* plane is built up from O—H⋯O and N—H⋯O hydrogen bonds.

## Related literature

For a related structure, see: Lalancette *et al.* (2001 ▶)
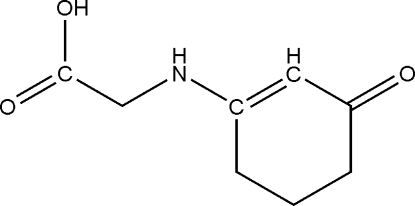


## Experimental

### 

#### Crystal data

C_8_H_11_NO_3_
*M* *_r_* = 169.18
Monoclinic, 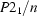

*a* = 5.138 (1) Å
*b* = 12.983 (3) Å
*c* = 12.345 (3) Å
β = 92.89 (3)°
*V* = 822.4 (3) Å^3^
*Z* = 4
Mo *K*α radiation
μ = 0.11 mm^−1^
*T* = 291 K
0.35 × 0.31 × 0.23 mm

#### Data collection

Rigaku R-AXIS RAPID diffractometer
Absorption correction: multi-scan (*ABSCOR*; Higashi, 1995 ▶) *T*_min_ = 0.964, *T*_max_ = 0.977
7743 measured reflections
1854 independent reflections
1461 reflections with *I* > 2σ(*I*)
*R*_int_ = 0.032

#### Refinement

*R*[*F* ^2^ > 2σ(*F* ^2^)] = 0.041
*wR*(*F* ^2^) = 0.117
*S* = 1.13
1854 reflections
127 parameters
8 restraints
H atoms treated by a mixture of independent and constrained refinement
Δρ_max_ = 0.18 e Å^−3^
Δρ_min_ = −0.19 e Å^−3^

### 

Data collection: *RAPID-AUTO* (Rigaku, 1998 ▶); cell refinement: *RAPID-AUTO*; data reduction: *CrystalClear* (Rigaku/MSC, 2002 ▶); program(s) used to solve structure: *SHELXS97* (Sheldrick, 2008 ▶); program(s) used to refine structure: *SHELXL97* (Sheldrick, 2008 ▶); molecular graphics: *SHELXTL* (Sheldrick, 2008 ▶); software used to prepare material for publication: *SHELXL97*.

## Supplementary Material

Crystal structure: contains datablocks I, global. DOI: 10.1107/S1600536809039646/ng2648sup1.cif

Structure factors: contains datablocks I. DOI: 10.1107/S1600536809039646/ng2648Isup2.hkl

Additional supplementary materials:  crystallographic information; 3D view; checkCIF report

## Figures and Tables

**Table 1:** Hydrogen-bond geometry (Å, °)

*D*—H⋯*A*	*D*—H	H⋯*A*	*D*⋯*A*	*D*—H⋯*A*
N1—H1⋯O1^i^	0.853 (9)	2.190 (10)	3.0266 (17)	166.9 (17)
O2—H2⋯O3^ii^	0.862 (10)	1.676 (10)	2.5369 (16)	176 (2)

Symmetry codes: (i) 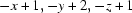; (ii) 
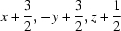
.

**Fig. 1.**
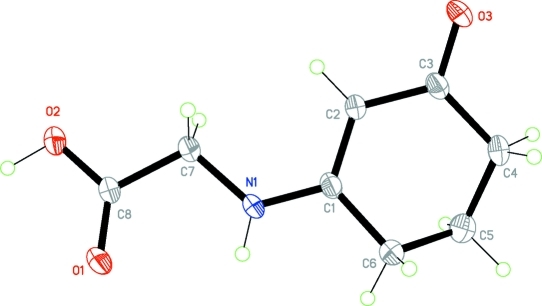
The molecular structure of the title compound, showing displacement ellipsoids at the 30% probability level for non-H atoms.

**Fig. 2.**
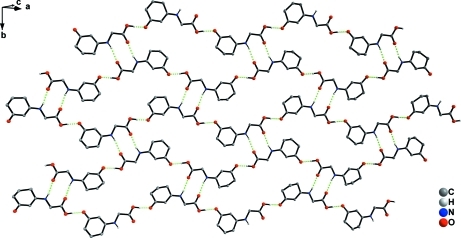
A partial packing view, showing the two-dimensional network. Dashed lines indicate the hydrogen-bonding interactions and no involving H atoms have been omitted.

## supplementary crystallographic information

### Comment

As shown in the scheme, the title compound is a resonance body of the (*E*)-2-(3-oxocyclohexylideneamino)acetic acid, which can be verified by the existence of the shorter single C—C bond distance and the emergence of the imino group (Figure 1). The C5 atom of the cyclohexane is disordered over two positions with site occupation 0.84 and 0.16 for C5, C5' and their appended H atoms, respectively.

In the crystal structure, a two-dimensional supramolecular network is built up by O—H···O and N—H···O hydrogen bonds between the ketone, the imino group and the carboxyl, along *ac* plane (Table 1, Figure 2).

### Experimental

Title compound was prepared from 1,3-cyclohexanedione (11.2 g, 0.1 mol) and 2-aminoacetic acid (7.5 g, 0.1 mol) in 100 ml DMSO solution at 100 °C for 24 h. Needle crystals for X-ray diffraction analysis were produced from a ethanol and cyclohexane mixed solution.

### Refinement

H atoms bonded to C atoms were placed in calculated positions and treated as riding on their parent atoms, with C—H = 0.93 Å (C2—H2A), C—H = 0.97 Å (methylene), and with *U*_iso_(H) = 1.2*U*_eq_(C). H atoms of the carboxyl group and imino group were located in a difference Fourier map and were freely refined with O—H = 0.85 Å, N—H = 0.85 Å. The C5 atom of the cyclohexane is disordered over two positions with site occupation 0.84 and 0.16 for C5, C5' and their appended H atoms, respectively.

### Crystal data

**Table:**

C_8_H_11_NO_3_	*F*(000) = 360
*M**_r_* = 169.18	*D*_x_ = 1.366 Mg m^−^^3^
Monoclinic, *P*2_1_/*n*	Mo *K*α radiation, λ = 0.71073 Å
Hall symbol: -P 2yn	Cell parameters from 6177 reflections
*a* = 5.138 (1) Å	θ = 3.1–27.5°
*b* = 12.983 (3) Å	µ = 0.11 mm^−^^1^
*c* = 12.345 (3) Å	*T* = 291 K
β = 92.89 (3)°	Block, colorless
*V* = 822.4 (3) Å^3^	0.35 × 0.31 × 0.23 mm
*Z* = 4	

### Data collection

**Table:**

Rigaku R-AXIS RAPID diffractometer	1854 independent reflections
Radiation source: fine-focus sealed tube	1461 reflections with *I* > 2σ(*I*)
graphite	*R*_int_ = 0.032
ω scans	θ_max_ = 27.5°, θ_min_ = 3.1°
Absorption correction: multi-scan (*ABSCOR*; Higashi, 1995)	*h* = −6→5
*T*_min_ = 0.964, *T*_max_ = 0.977	*k* = −16→16
7743 measured reflections	*l* = −15→15

### Refinement

**Table:**

Refinement on *F*^2^	Primary atom site location: structure-invariant direct methods
Least-squares matrix: full	Secondary atom site location: difference Fourier map
*R*[*F*^2^ > 2σ(*F*^2^)] = 0.041	Hydrogen site location: inferred from neighbouring sites
*wR*(*F*^2^) = 0.117	H atoms treated by a mixture of independent and constrained refinement
*S* = 1.13	*w* = 1/[σ^2^(*F*_o_^2^) + (0.0593*P*)^2^ + 0.1182*P*] where *P* = (*F*_o_^2^ + 2*F*_c_^2^)/3
1854 reflections	(Δ/σ)_max_ < 0.001
127 parameters	Δρ_max_ = 0.18 e Å^−^^3^
8 restraints	Δρ_min_ = −0.19 e Å^−^^3^

### Special details

**Table:**

Geometry. All e.s.d.'s (except the e.s.d. in the dihedral angle between two l.s. planes) are estimated using the full covariance matrix. The cell e.s.d.'s are taken into account individually in the estimation of e.s.d.'s in distances, angles and torsion angles; correlations between e.s.d.'s in cell parameters are only used when they are defined by crystal symmetry. An approximate (isotropic) treatment of cell e.s.d.'s is used for estimating e.s.d.'s involving l.s. planes.
Refinement. Refinement of *F*^2^ against ALL reflections. The weighted *R*-factor *wR* and goodness of fit *S* are based on *F*^2^, conventional *R*-factors *R* are based on *F*, with *F* set to zero for negative *F*^2^. The threshold expression of *F*^2^ > σ(*F*^2^) is used only for calculating *R*-factors(gt) *etc*. and is not relevant to the choice of reflections for refinement. *R*-factors based on *F*^2^ are statistically about twice as large as those based on *F*, and *R*- factors based on ALL data will be even larger.

### Fractional atomic coordinates and isotropic or equivalent isotropic displacement parameters (Å^2^)

**Table:**

	*x*	*y*	*z*	*U*_iso_*/*U*_eq_	Occ. (<1)
C5'	−0.308 (3)	1.0310 (8)	0.2164 (12)	0.043 (4)	0.135 (6)
H5'1	−0.4453	1.0472	0.2648	0.052*	0.135 (6)
H5'2	−0.2880	1.0900	0.1693	0.052*	0.135 (6)
C5	−0.1653 (5)	1.01745 (14)	0.16773 (17)	0.0444 (7)	0.865 (6)
H5A	−0.0283	0.9987	0.1202	0.053*	0.865 (6)
H5B	−0.2226	1.0867	0.1489	0.053*	0.865 (6)
C1	0.0056 (2)	0.90870 (10)	0.31865 (10)	0.0301 (3)	
C2	−0.1357 (3)	0.82688 (10)	0.27717 (11)	0.0331 (3)	
H2A	−0.0941	0.7608	0.3015	0.040*	
C3	−0.3418 (3)	0.83987 (10)	0.19878 (10)	0.0323 (3)	
C4	−0.3913 (3)	0.94381 (12)	0.15005 (13)	0.0439 (4)	
H4A	−0.5440	0.9734	0.1811	0.053*	
H4B	−0.4292	0.9360	0.0727	0.053*	
C6	−0.0589 (3)	1.01603 (11)	0.28306 (13)	0.0445 (4)	
H6A	0.0968	1.0583	0.2897	0.053*	
H6B	−0.1866	1.0450	0.3297	0.053*	
C7	0.2932 (3)	0.79797 (11)	0.43069 (11)	0.0348 (3)	
H7A	0.3407	0.7560	0.3698	0.042*	
H7B	0.1534	0.7633	0.4661	0.042*	
C8	0.5245 (3)	0.80897 (10)	0.50935 (10)	0.0324 (3)	
H1	0.274 (3)	0.9517 (10)	0.4179 (13)	0.053 (5)*	
H2	0.762 (3)	0.7271 (19)	0.5786 (16)	0.089 (8)*	
N1	0.2035 (2)	0.89704 (9)	0.39184 (9)	0.0350 (3)	
O1	0.5998 (2)	0.89070 (8)	0.54410 (10)	0.0549 (4)	
O2	0.6287 (2)	0.72005 (8)	0.53416 (8)	0.0425 (3)	
O3	−0.4844 (2)	0.76566 (8)	0.16931 (9)	0.0468 (3)	

### Atomic displacement parameters (Å^2^)

**Table:**

	*U*^11^	*U*^22^	*U*^33^	*U*^12^	*U*^13^	*U*^23^
C5'	0.044 (7)	0.025 (5)	0.059 (7)	0.005 (4)	−0.011 (5)	0.004 (4)
C5	0.0481 (13)	0.0335 (9)	0.0494 (12)	−0.0016 (8)	−0.0183 (10)	0.0112 (7)
C1	0.0275 (6)	0.0328 (7)	0.0291 (6)	0.0012 (5)	−0.0077 (5)	−0.0014 (5)
C2	0.0330 (7)	0.0294 (7)	0.0353 (7)	−0.0022 (5)	−0.0134 (5)	0.0034 (5)
C3	0.0288 (6)	0.0369 (7)	0.0302 (6)	−0.0024 (5)	−0.0083 (5)	−0.0024 (5)
C4	0.0399 (8)	0.0430 (8)	0.0464 (8)	0.0020 (6)	−0.0205 (6)	0.0053 (6)
C6	0.0435 (8)	0.0295 (7)	0.0579 (9)	0.0025 (6)	−0.0221 (7)	−0.0043 (6)
C7	0.0318 (7)	0.0362 (7)	0.0350 (7)	−0.0053 (5)	−0.0132 (6)	0.0044 (5)
C8	0.0305 (6)	0.0356 (7)	0.0300 (6)	−0.0006 (5)	−0.0093 (5)	0.0010 (5)
N1	0.0328 (6)	0.0323 (6)	0.0379 (6)	−0.0032 (5)	−0.0172 (5)	−0.0015 (4)
O1	0.0560 (7)	0.0383 (6)	0.0662 (7)	0.0015 (5)	−0.0378 (6)	−0.0078 (5)
O2	0.0438 (6)	0.0385 (6)	0.0431 (6)	0.0038 (4)	−0.0195 (5)	0.0021 (4)
O3	0.0441 (6)	0.0423 (6)	0.0510 (6)	−0.0104 (4)	−0.0254 (5)	−0.0008 (5)

### Geometric parameters (Å, °)

**Table:**

C5'—C4	1.448 (12)	C3—C4	1.494 (2)
C5'—C6	1.500 (12)	C4—H4A	0.9700
C5'—H5'1	0.9700	C4—H4B	0.9700
C5'—H5'2	0.9700	C6—H6A	0.9700
C5—C6	1.499 (2)	C6—H6B	0.9700
C5—C4	1.511 (2)	C7—N1	1.4403 (17)
C5—H5A	0.9700	C7—C8	1.5030 (18)
C5—H5B	0.9700	C7—H7A	0.9700
C1—N1	1.3344 (17)	C7—H7B	0.9700
C1—C2	1.3710 (18)	C8—O1	1.2013 (17)
C1—C6	1.4931 (19)	C8—O2	1.3025 (17)
C2—C3	1.4082 (18)	N1—H1	0.853 (9)
C2—H2A	0.9300	O2—H2	0.862 (10)
C3—O3	1.2533 (16)		
			
C4—C5'—C6	115.4 (8)	C5'—C4—H4B	132.6
C4—C5'—H5'1	108.4	C3—C4—H4B	108.9
C6—C5'—H5'1	108.4	C5—C4—H4B	108.9
C4—C5'—H5'2	108.4	H4A—C4—H4B	107.7
C6—C5'—H5'2	108.4	C1—C6—C5	110.84 (13)
H5'1—C5'—H5'2	107.5	C1—C6—C5'	116.9 (4)
C6—C5—C4	111.72 (16)	C5—C6—C5'	38.4 (6)
C6—C5—H5A	109.3	C1—C6—H6A	109.5
C4—C5—H5A	109.3	C5—C6—H6A	109.5
C6—C5—H5B	109.3	C5'—C6—H6A	130.6
C4—C5—H5B	109.3	C1—C6—H6B	109.5
H5A—C5—H5B	107.9	C5—C6—H6B	109.5
N1—C1—C2	122.41 (12)	C5'—C6—H6B	72.0
N1—C1—C6	117.07 (12)	H6A—C6—H6B	108.1
C2—C1—C6	120.52 (12)	N1—C7—C8	111.08 (11)
C1—C2—C3	121.96 (12)	N1—C7—H7A	109.4
C1—C2—H2A	119.0	C8—C7—H7A	109.4
C3—C2—H2A	119.0	N1—C7—H7B	109.4
O3—C3—C2	121.05 (13)	C8—C7—H7B	109.4
O3—C3—C4	119.49 (12)	H7A—C7—H7B	108.0
C2—C3—C4	119.46 (12)	O1—C8—O2	125.30 (13)
C5'—C4—C3	116.0 (5)	O1—C8—C7	122.99 (12)
C5'—C4—C5	38.9 (6)	O2—C8—C7	111.71 (11)
C3—C4—C5	113.54 (12)	C1—N1—C7	123.16 (11)
C5'—C4—H4A	71.8	C1—N1—H1	117.2 (13)
C3—C4—H4A	108.9	C7—N1—H1	119.6 (13)
C5—C4—H4A	108.9	C8—O2—H2	111.1 (17)

### Hydrogen-bond geometry (Å, °)

**Table:**

*D*—H···*A*	*D*—H	H···*A*	*D*···*A*	*D*—H···*A*
N1—H1···O1^i^	0.85 (1)	2.19 (1)	3.0266 (17)	167 (2)
O2—H2···O3^ii^	0.86 (1)	1.68 (1)	2.5369 (16)	176 (2)

Symmetry codes: (i) −*x*+1, −*y*+2, −*z*+1; (ii) *x*+3/2, −*y*+3/2, *z*+1/2.
